# Cholinergic centro-cingulate network in Parkinson disease and normal aging

**DOI:** 10.18632/aging.205209

**Published:** 2023-10-27

**Authors:** Nicolaas I. Bohnen, Sygrid van der Zee, Roger Albin

**Affiliations:** 1 Department of Radiology, University of Michigan, Ann Arbor, MI 48105, USA; 2 Neurology Service and GRECC, Veterans Administration Ann Arbor Healthcare System, Ann Arbor, MI 48105, USA; 3 Morris K. Udall Center of Excellence for Parkinson's Disease Research, University of Michigan, Ann Arbor, MI 48109, USA; 4 Parkinson’s Foundation Center of Excellence, University of Michigan, Ann Arbor, MI 48109, USA; 5 Department of Neurology, University of Groningen, University Medical Center Groningen, Groningen, The Netherlands; 6 Department of Neuropsychology, University of Groningen, University Medical Center Groningen, Groningen, The Netherlands; 7 Department of Neurology, University of Michigan, Ann Arbor, MI 48109, USA

**Keywords:** centro-cingulate network, cholinergic, cognition, motor, Parkinson disease

## Abstract

Decreased cholinergic binding within the recently identified centro-cingulate brain network robustly has been shown to robustly correlate with the severity of cognitive impairment in Parkinson disease (PD). This network with key hubs within the cingulum, operculum and peri-central cortical regions also correlates with elements of parkinsonian motor impairments, including postural instability and gait difficulties, such as falls or freezing. MRI neuroimaging studies have shown that the anterior midcingulate cortex is a key node for cognitive aspects of movement generation, i.e., intentional motor control. Recent evidence also suggests a novel aspect of organization of primary motor cortex, describing “effector” regions for fine movement control intercalated with interlinked “inter-effector” regions devoted to whole-body control. A distinguishing feature of inter-effector regions is tight linkage to the cingular and opercular regions. Such inter-effector regions have been proposed to be part of a greater somato-cognitive action network necessary for integration of goals and movement.

Recent evidence also points to vulnerabilities of cholinergic nerve terminals in the centro-cingulate network in older non-PD adults. These features of normal aging underscore that cortical cholinergic terminal losses in age-associated neurodegenerative disorders are likely not exclusively the result of disease-specific etiologies but also related to otherwise normal aging. Practical implications of this overlap are that addressing disease-specific and general aging etiologies involved in neurodegeneration, may be of benefit in age-associated neurodegenerative disorders where significant cholinergic systems degeneration is present.

## INTRODUCTION

We recently reported novel data-driving findings suggesting that cholinergic innervation deficits in centro-cingulate brain regions may be an important contributor to cognitive impairments in PD [1]. Using the spatially resolute presynaptic vesicular acetylcholine transporter PET radiotracer [^18^F]Fluoroethoxy benzovesamicol ([^18^F]FEOBV) and principal component analysis, we identified a specific component localizing to cingulate cortices, particularly anterior to mid-cingulum, bilateral precuneus, bilateral paracentral lobules, bilateral pericentral (pre- and postcentral cortices gyri), bilateral mid to superior mesofrontal regions, right insula (partially), bilateral upper medial frontopolar cortex (upper medial part of Brodmann area 10), bilateral dorsomedial prefrontal cortex (Brodmann areas 8 and 9), and bilateral frontotemporal operculum. Among the seven identified principal components, this specific component had the most robust associations with variations in a broad range of cognitive functions: global cognition, memory, execution, attention and language.

The topography of this centro-cingulate component (midline cingulum structures combined with bilateral peri-central regions, right insula, and mid to upper mesofrontal regions) may suggest involvement of cortical networks subserving higher order cognitive control functions. In particular, the centro-cingulate component topography significantly overlaps with the functional MRI-identified cingulo-opercular and salience networks. The cingulo-opercular task control network (COTC) is implicated in important cognitive functions, including maintenance of alertness, task set maintenance, and salience detection of stimuli. Basal forebrain cholinergic afferents are likely important modulators of these cognitive control and salience attentional networks. Further support for an important role of these centro-cingulate cholinergic afferents comes from converging cognitive domain-specific analysis showing partially shared topography for memory, cognitive and executive function domains [2]. Zaborszky et al. demonstrated that basal forebrain cholinergic neurons give rise to collateralized but restricted projections to interconnected cortical regions [3]. It is plausible that our principal component analysis identified basal forebrain cholinergic neurons integrated into central functions of the cingulo-opercular and salience networks.

Our analysis from the same dataset also showed a significant relationship between centro-cingulate cholinergic afferents and standard measures of PD global motor impairments ratings and disease severity staging. We previously reported that assessments of non-episodic postural instabilities and gait difficulties had significant correlations with the integrity of anterior cingulate cholinergic afferents in PD [4]. The topography of cholinergic system changes associated with episodic mobility disturbances, such as falls and freezing of gait, in PD also partially overlaps with the centro-cingulate cholinergic afferent component [5]. The rostral cingulate cortex is associated with a multitude of cognitive control functions. One functional MRI study suggested that the anterior midcingulate cortex is a key node for cognitive aspects of movement generation, i.e., intentional motor control [6]. Connectivity analysis found that the “cognition” domain showed higher convergence of activity in supramodal association areas of prefrontal cortex and anterior insula with “action” domain yielding higher convergence of activity in somatosensory and premotor areas. These authors concluded that the anterior midcingulate cortex plays a role in the intentional generation of movements and timing of movement. In a recent, impressive application of MRI methods, Gordon et al. identified a novel aspect of organization of primary motor cortex (M1), describing “effector” regions for fine movement control intercalated with interlinked “inter-effector” regions devoted to whole-body control [7]. A distinguishing feature of inter-effector regions is tight linkage to the cingulo-opercular network. Gordon et al. propose that the inter-effector regions are part of a greater somato-cognitive action network (SCAN) necessary for integration of goals and movement. Taken together, these observations and our recent work suggests that the centro-cingulate cholinergic afferent component has relevance for PD not only for cognitive control, but also for crucial aspects of integrative intentional motor control and action. This inference is consistent with prior data indicating that cholinergic terminal deficits in the paracentral lobule are associated with abnormalities in distal limb bradykinesia ratings in PD [4].

The SCAN concept and the potential role of cholinergic terminal deficits within this extended network is also relevant to maintenance of postural stability. Specific overlap of cholinergic terminal deficits with fall history in PD includes changes in the right frontotemporal operculum and insula, whereas the mid-cingulum overlaps with cholinergic afferent deficits found in freezing of gait. Saliency, attentional, and motor integration play a key mechanistic role in falls. These findings are consistent with key roles for centro-cingulate-SCAN dysfunction in the pathophysiology of episodic PD motor deficits and underscore the attentional, broader cognitive motor integration aspects of postural instability and gait difficulties in PD. Collectively, these findings indicate that centro-cingulate cholinergic afferents are relevant for cognitive and motor impairments in PD, especially affecting function relying on saliency cue detection and attentional, cognitive motor integration, and intentional motor control.

Our principal component analyses showed a significant relationship between centro-cingulate cholinergic afferent changes and age in our PD subjects [1]. Recently, we reported the effects of aging and the topography of cerebral cholinergic deficits in neurologically intact adults [8]. Examining the topography of age-related cholinergic denervation, we found declines in centro-cingulate cholinergic afferents with nearly all regions examined vulnerable to the effects of aging. The only exceptions were the bilateral mid-mesofrontal regions. Table 1 shows a summary of overlapping regions for specific cognitive domains, motor impairments in PD, and normal aging effects for the major centro-cingulate cholinergic afferent regions.

**Table 1:** Summary overview of partially shared cholinergic centro-cingulate key regions with topographic system changes associating with cognition and motor impairments in PD and normal aging.

	**Cognition and motor impairments in PD and normal aging**							
**Key regions of the centro-cingulate network**	**Memory domain scores**	**Executive function domain scores**	**Attention domain scores**	**Non-episodic balance and gait changes**	**Distal limb bradykinesia**	**Falls**	**Freezing of gait**	**Normal aging**
Caudal anterior cingulum (L)	✔	✔	✔	✔		✔	✔	✔
Mid cingulum (L)	✔	✔	✔					✔
Posterior cingulum & precuneus (B; partially)	✔	✔						✔
Isthmus cingulum (BA 30, part of the retrosplenial cingulum, B)	✔							✔
Insula (partially, R)	✔ B	✔ B	✔ L>R			✔	✔	✔
Frontotemporal operculum (B)	✔ B	✔ B	✔B			✔R	✔R	✔ B
Upper medial frontopolar cortex (upper part of BA 10, B)	✔ R					✔R	✔R	
Dorsomedial prefrontal cortex (BA 8 and 9, B)	✔ B	✔ R				✔R	✔R	✔
Paracentral lobule (B)	✔ B	✔ R			✔			✔
Pre- and post-central cortex (B)	✔ B	✔						✔

Abbreviations: BA: Brodmann area; B: bilateral; L: left; R: right.

These features of normal aging underscore that cortical cholinergic terminal losses in age-associated neurodegenerative disorders are likely not exclusively the result of disease-specific etiologies but also related to otherwise normal aging. As with nigrostriatal dopaminergic projection degeneration, normal aging effects may make cholinergic projection neurons particularly sensitive to the cellular pathologies driving PD and other neurodegenerative disorders. Practical implications of this overlap are that addressing disease-specific and general aging etiologies involved in neurodegeneration, may be of benefit in neurodegenerative disorders where significant cholinergic systems degeneration is present.
